# Sleep-Wake Cycle Dysfunction in the TgCRND8 Mouse Model of Alzheimer’s Disease: From Early to Advanced Pathological Stages

**DOI:** 10.1371/journal.pone.0130177

**Published:** 2015-06-15

**Authors:** Jessica Colby-Milley, Chelsea Cavanagh, Sonia Jego, John C. S. Breitner, Rémi Quirion, Antoine Adamantidis

**Affiliations:** 1 Douglas Mental Health University Institute, Dept. of Psychiatry, McGill University, Montreal, Quebec, H4H 1R3, Canada; 2 Inselspital, Bern University Hospital, Bern University, Dept. of Neurology, Freiburgstrasse, 18, 3010 Bern, Switzerland; University of Oxford, UNITED KINGDOM

## Abstract

In addition to cognitive decline, individuals affected by Alzheimer’s disease (AD) can experience important neuropsychiatric symptoms including sleep disturbances. We characterized the sleep-wake cycle in the TgCRND8 mouse model of AD, which overexpresses a mutant human form of amyloid precursor protein resulting in high levels of β-amyloid and plaque formation by 3 months of age. Polysomnographic recordings in freely-moving mice were conducted to study sleep-wake cycle architecture at 3, 7 and 11 months of age and corresponding levels of β-amyloid in brain regions regulating sleep-wake states were measured. At all ages, TgCRND8 mice showed increased wakefulness and reduced non-rapid eye movement (NREM) sleep during the resting and active phases. Increased wakefulness in TgCRND8 mice was accompanied by a shift in the waking power spectrum towards fast frequency oscillations in the beta (14-20 Hz) and low gamma range (20-50 Hz). Given the phenotype of hyperarousal observed in TgCRND8 mice, the role of noradrenergic transmission in the promotion of arousal, and previous work reporting an early disruption of the noradrenergic system in TgCRND8, we tested the effects of the alpha-1-adrenoreceptor antagonist, prazosin, on sleep-wake patterns in TgCRND8 and non-transgenic (NTg) mice. We found that a lower dose (2 mg/kg) of prazosin increased NREM sleep in NTg but not in TgCRND8 mice, whereas a higher dose (5 mg/kg) increased NREM sleep in both genotypes, suggesting altered sensitivity to noradrenergic blockade in TgCRND8 mice. Collectively our results demonstrate that amyloidosis in TgCRND8 mice is associated with sleep-wake cycle dysfunction, characterized by hyperarousal, validating this model as a tool towards understanding the relationship between β-amyloid overproduction and disrupted sleep-wake patterns in AD.

## Introduction

Alzheimer’s disease (AD) is a neurodegenerative disorder with a rapidly growing worldwide prevalence and no effective disease-modifying treatments. Key neuropathological features of AD include β-Amyloid (Aβ) plaques, neurofibrillary tangles composed of hyperphosporylated tau protein, synaptic loss and widespread brain atrophy. In addition to memory loss, AD patients often experience important non-cognitive, neuropsychiatric symptoms, which include perturbations of the sleep-wake cycle [1–3].

Sleep disturbances occur in up to 40% of AD patients [4] and clinical manifestations of sleep-wake cycle dysfunction include difficulties falling asleep, fragmented sleep, early morning awakenings and excessive daytime sleepiness [4–6]. Specific changes in sleep architecture include decreased time spent in slow-wave, non-rapid eye movement (NREM) sleep, and decreased time spent in rapid eye movement (REM) sleep, the latter occurring at more advanced stages of the disease [7, 8]. Importantly, human studies increasingly suggest an early occurrence of sleep disturbances in the development of AD, and even show an association between disrupted sleep in cognitively normal individuals and increased risk of AD later in life [9–13]. Furthermore, research in AD mouse models has highlighted the potential for disrupted sleep-wake patterns to actively contribute to AD pathology, rather than solely existing as a corollary symptom of the disease [14–16]. Specifically, paradigms that promote wakefulness result in increased extracellular levels of soluble Aβ and increased deposition of Aβ into insoluble plaques [14]. Thus, identifying AD animal models that recapitulate the sleep-wake cycle patterns observed in AD will provide tools towards understanding the cause and progression of sleep dysfunction, which in turn, may have important implications for therapeutic intervention in AD patients.

The vigilance states of sleep and wakefulness are regulated by several diffusely projecting and highly interconnected neurotransmitter systems originating in the brainstem, hypothalamus, thalamus, and basal forebrain [17, 18]. Wakefulness is promoted by the combined excitatory actions of acetylcholine, noradrenaline, histamine, serotonin, dopamine, and orexin at widespread cortical, subcortical, and thalamic sites [18, 19]. Within this ascending arousal system, wakefulness is further enhanced by potentiating excitatory effects between wake-promoting neuronal populations, as well as by the inhibitory actions of a number of these neurotransmitters, including noradrenaline and acetylcholine, at sleep-promoting regions [18]. In turn, NREM sleep is thought to result from inhibitory actions of γ-aminobutyric acid (GABA) and galanin expressing neurons of the hypothalamic preoptic area on wake-promoting neuronal populations, and ultimately, the occurrence of slow oscillations in the thalamocortical network [18]. The mutually inhibitory connections that exist between wake and sleep promoting neuronal populations allow for a sharp transition between sleep and wakefulness and reduce the occurrence of intermediate states of arousal [20].

In addition to the interplay between the sleep and wake promoting neurotransmitter systems described, another driving force affecting the sleep-wake cycle is the homeostatic process of sleep regulation. Throughout the active period, as wakefulness persists, there is a gradual build-up of sleep pressure and a demand for sleep recovery [21]. This build-up will affect sleep in the subsequent rest period, resulting in a compensatory increase in sleep duration and NREM sleep intensity, the latter indexed by NREM delta power [22].

Given the widespread nature of neuronal injury and loss in AD, virtually all of the neurotransmitter systems regulating sleep and wakefulness are affected to some degree during the course of the disease [23]. Of these, the noradrenergic locus coeruleus nucleus, the main source of noradrenaline in the brain [24], undergoes severe neurodegeneration in AD [25–27], experiencing as great as 80% neuronal loss [28]. Furthermore, when noradrenergic and cholinergic cell loss was examined concurrently in AD, neurodegeneration from the locus coeruleus was greater than cholinergic loss in the nucleus basalis of the basal forebrain [29]. Interestingly, there is evidence supporting a compensatory increase in noradrenergic activity in response to locus coeruleus cell loss in AD [30–32]. Furthermore, It has been proposed that compensatory increases in activity within the noradrenergic system may contribute the behavioural and psychological symptoms of agitation and aggression in AD [33].

The TgCRND8 mouse model of AD expresses a double mutant form of the human amyloid precursor protein (APP) 695, resulting in early-onset deposition of Aβ, with plaques appearing in 100% of mice by 3 months of age and neuritic pathology appearing at 5 months of age [34]. In the current study, we first sought out to characterize sleep-wake architecture under baseline conditions in the TgCRND8 mouse model of AD using polysomnographic recordings in freely-moving mice at 3, 7 and 11 months of age. Our second objective was to assess the homeostatic process of sleep regulation in TgCRND8 mice. Next, we quantified region-specific Aβ_42_ levels in key brain structures controlling sleep-wake states. Finally, we investigated whether blockade of noradrenergic transmission could normalize sleep levels in TgCRND8 mice.

## Methods and Materials

### Animal model

TgCRND8 mice carry a double mutant form (Swedish (K670N/M671L) and Indiana (V717F) mutations) of human APP 695 on a C57BL/6-C3H/HeJ hybrid background [34]. The expression of the transgene is under the control of the syrian hamster prion promoter [34]. Only male, heterozygous TgCRND8 mice were used in all experimental groups. Age groups studied included 3 months ± 15 days, 7 months ± 30 days, 11 months ±15 days for baseline polysomnographic recordings; 3 months ± 15 days for sleep deprivation; 3 months ±18 days, 7 months ±15 days, 11 months ±30 days for Aβ_42_ quantification and 3.5 months for polysomnographic recordings following treatment with Prazosin. Independent cohorts of mice were used in each experiment (including age groups), with the exception of the 3-month old group used for baseline recordings followed by total sleep deprivation. The mice were housed under constant temperature (22°C) under a 12–12 hr light-dark cycle (light onset: 8:00 am) with food and water accessible *ad libitum*.

### Ethics Statement

All animal protocols were carried out in accordance with the National Institute of Health’s “Guide for the care and use of Laboratory Animals” and were approved by McGill University’s Animal Care Committee and the Canadian Council of Animal Care (protocol number: 3589). For experiments involving brain tissue collection, mice were first anaesthetized with isoflurane, and euthanized by decapitation. For experiments involving stereotaxic brain surgery, isoflurane was used for anaesthesia.

### Surgery

In order to collect polysomnographic data, animals were chronically implanted with electroencephalogram/electromyogram (EEG/EMG) electrodes as previously described [35]. Briefly, Cortical electrodes were inserted into the skull bilaterally above the frontal (2mm lateral and anterior to bregma) and parietal cortices (2mm lateral to the midline at the midpoint between bregma and lambda). All cortical electrodes were fixed to the skull via SuperBond (Sun Medical Co., Shiga, Japan) and acrylic cement. Following surgery, animals were housed individually in their home cage for a recovery period of 15 days. Six to 10 days before polysomnographic recordings, the mice were transferred to a recording room and placed in individual recording cages where their electrodes were connected to collecting cables for habituation.

### Polysomnographic recording and data acquisition

Twenty-four hour baseline recordings were performed beginning at 8:00 PM. EEG and EMG signals from electrodes were amplified (Grass Instruments, USA), digitized at a sampling rate of 512 Hz and collected on a PC within the recording room using *VitalRecorder* (Kissei Comtec Co. Ltd, Japan), as previously described [35]. EEG and EMG signals were processed with low and high pass filters of 0.5–80 Hz and 20–40 Hz, respectively. None of the mice analyzed showed any EEG or EMG artifacts over 24 hr. polysomnographic recordings. The polysomnographic recordings were visually scored offline using *SleepSign* (Kissei Comtec Co. Ltd, Japan), per 5 second epoch window, as either wake, NREM sleep or REM sleep, as previously described [35]. Briefly, behavioural state was determined according to the EEG spectra power and the following criteria:

-Wake: High frequency low-amplitude EEG oscillations accompanied by constant EMG activity with phasic bursts.
-NREM sleep: Low frequency, high amplitude EEG oscillations with an increase in slow delta wave activity (0.5–4.5 Hz) and a loss of phasic muscle activity.
-REM sleep: High frequency, low amplitude EEG oscillations with typical regular theta rhythm (6–10 Hz) and a flat EMG.

Epochs were consistently scored based on the following criteria: if an epoch showed the signature EEG and EMG features of wake, NREM, or REM sleep for > 50% of the epoch, it was scored as the corresponding vigilance state. Notably, this criteria permitted for the detection of microarousal events < 5 seconds.

The scored EEG signals were subjected to fast Fourier transform (FFT) in SleepSign (Kissei Comtec Co. Ltd, Japan) to generate power spectra during wake, NREM and REM across the light and dark period. A 5-second epoch window (frequency resolution: 0.2 Hz) with a Hanning filter and FFT size of 4096 were used to calculate the FFT. Power spectra were calculated from 0.125–100 Hz with a 0.125 Hz bin width. In order to minimize local variations in oscillation amplitude between animals, which may arise due to slight differences in the exact placement of electrodes, power values were normalized to the sum of all power values (V^2^) from 0.125–100 Hz. Power values were then calculated for the following frequency bands: delta (0.5–4.5 Hz), theta (6–10 Hz), alpha (10–14 Hz), beta (14–20 Hz), low gamma (20–50 Hz).

### Sleep deprivation

Polysomnographic recordings on 3-month-old TgCRND8 were performed for 48 hrs and consisted of an initial 24 hr baseline recording (8:00 PM-8:00 PM) and a 6 hr period of total sleep deprivation (TSD) beginning at the subsequent light phase (8:00 AM-2:00 PM) using the gentle handling technique, as previously described [36]. Following TSD, the recording was continued from 2:00 PM until 8:00 PM for assessment of sleep recovery.

### Region-specific quantification of total Aβ_42_

The hypothalamus, thalamus, prefrontal cortex and brainstem of 3, 7 and 11 month-old TgCRND8 mice were dissected in phosphate buffer saline on a 4°C surface and stored at -80°C. Total Aβ was extracted according to the extraction procedure from Aβ_42_ ELISA kit (KHB3442, Invitrogen Life Technologies, CA, USA). Briefly, the wet mass of each sample was taken and homogenized with 5M guanidine hydrochloride (G9284, Sigma-Aldrich, Mo, USA). Samples were then diluted with BSAT-DPBS, supplemented with 1X protease inhibitor cocktail (539131, Calbiochem, CA, USA). A protein assay was performed to determine mg of total protein/ml of extracted sample via a bicinchoninic acid protein assay (23227, Thermo Scientific, IL, USA). The Aβ_42_ ELISA was then performed according to the assay procedure notes (KHB3442, Invitrogen Life Technologies, CA, USA), with an overnight incubation at 4°C. Values obtained from the Aβ_42_ ELISA (pg/ml) were normalized to the total protein content of each sample (mg/ml).

### Prazosin treatment

Prazosin hydrochloride (P7791, Sigma-Aldrich, Mo, USA) was suspended in sterile ddH_2_0 and sonicated to obtain complete dissolution. 3.5-month-old TgCRND8 mice chronically implanted with EEG/EMG electrodes were administered an intraperitoneal injection of sterile ddH_2_0 (vehicle) each day at 10:00 AM for a period of 4 days in order to habituate them to the method of drug delivery. Treatment was performed in the polysomnographic recording room. On the last day of habituation, mice were weighed. Following habituation, over four consecutive days, TgCRND8 mice received doses of vehicle, 1 mg/kg, 2 mg/kg or 5 mg/kg of prazosin hydrochloride at 10:00 AM and polysomnographic recordings were collected for the remaining duration of the light phase. Analysis of sleep-wake states following prazosin administration revealed an effect in the first two hours following treatment, in accordance with its half-life (2–3 hrs).

### Statistical analysis

#### Experiment 1- Baseline polysomnographic recordings

Baseline data collected from 24 hr polysomnographic recordings in 3, 7 and 11-month old non-transgenic (NTg) and TgCRND8 was analyzed using two-way ANOVA (genotype and age both treated as between factors given that the three age groups represented different cohorts of mice). Sample sizes are as follows: 3 months NTg n = 7, Tg n = 8, 7 months NTg n = 5, Tg n = 7, 11 months NTg n = 6, Tg n = 4.

#### Experiment 2- Sleep deprivation

Sleep deprivation data were analyzed using a mixed design two-way ANOVA with genotype as a between factor and time period as a within factor. Sample sizes are as follows: NTg n = 7, Tg n = 9.

#### Experiment 3- Quantification of Aβ_42_

Levels of Aβ_42_ were analyzed by one-way ANOVA comparing the different brain regions (within factor) at a given age (3, 7 and 11 month-old TgCRDN8). Sample sizes are as follows: 3 months n = 7, 7 months n = 9, 11 months n = 7.

#### Experiment 4- Prazosin treatment

Vigilance state durations following treatment with prazosin were analyzed via two-way ANOVAs comparing each dose (1, 2 and 5 mg/kg) to vehicle. Sample sizes are as follows: NTg vehicle n = 11, NTg 1, 2 and 5 mg/kg n = 8, Tg vehicle n = 11, Tg 1 and 2 mg/kg n = 9, Tg 5 mg/kg n = 6.

For all statistical tests performed, significance was set at *P*<0.05. Significant main effects were investigated using Tukey’s multiple comparison test and significant interactions were assessed using the simple effects analysis unless otherwise stated. GraphPad Prism 5.0a and Datasim software were used for statistical analysis.

## Results

### TgCRND8 mice display sleep-wake cycle disruption at early and advanced pathological stages

**Fig 1:**
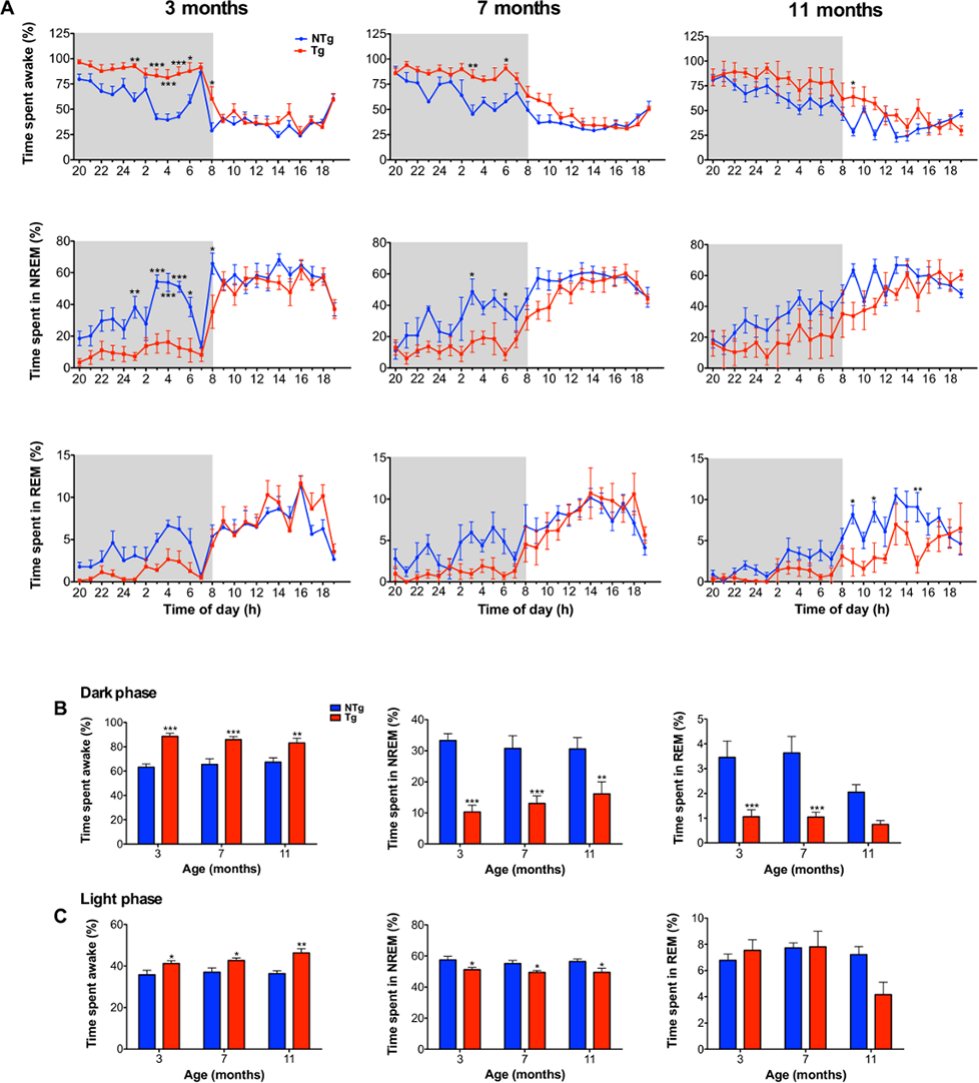
TgCRND8 mice show sleep-wake cycle disruption at early and advanced pathological stages. Hourly time courses (**A**) and cumulative percent duration (**B** and **C**) of wake, NREM and REM sleep across the light and dark phases at 3 (NTg n = 7, Tg n = 8), 7 (NTg n = 5, Tg n = 7) and 11(NTg n = 6, Tg n = 4) months of age. (**B)** During the dark phase, 3, 7 and 11-month-old TgCRND8 spend more time awake and less time in NREM sleep in comparison to NTg. Three and 7-month-old TgCRND8 mice also show a significant decrease in the percent time spent in REM sleep during the dark phase in comparison to NTg. (**C)** During the light phase, 3, 7 and 11-month-old TgCRND8 spend more time awake and less time in NREM sleep in comparison to NTg. Total time spent in REM sleep did not differ significantly between TgCRND8 and NTg during the light phase, at all ages studied. Error bars represent SEM. Panel **A** was analyzed by two-way ANOVA, followed by Bonferroni test for multiple comparisons. Panels **B** and **C** were analyzed by two-way ANOVA, followed by Tukey’s post-hoc test, * *P*<0.05, ** *P*<0.01, ***P<0.001.

To measure possible alterations in sleep-wake cycle architecture at early and advanced stages of amyloidosis, we recorded EEG and EMG signals from TgCRND8 mice at 3, 7 and 11 months of age. Polysomnographic recordings were analyzed to quantify the total time spent in wake, NREM sleep and REM sleep during the dark phase (DP) and light phase (LP) (Fig 1). It is of note that circadian fluctuation was intact in TgCRND8 mice as shown by a significantly greater time spent awake and less time spent in NREM and REM sleep during the dark versus the light phase at 3 (wake: *P*<0.001, NR: *P*<0.001, R: *P*<0.001), 7 (wake: *P*<0.001, NR: *P*<0.001, R: *P*<0.001), and 11 months of age (wake: *P*<0.01, NR: *P*<0.01, R, *P*<0.05) (data not shown).

During the dark phase (i.e active phase in mice), TgCRND8 mice spent a significantly greater amount of time awake in comparison to NTg at 3 (+25.4%; *P*<0.001), 7 (+20.3%; *P*<0.001) and 11 months of age (+15.8%; *P*<0.01) (main effect of genotype: *F*_1,31_ = 60.0, *P*<0.001, Fig 1B). TgCRND8 also showed a significant decrease in time spent in NREM sleep in comparison to NTg at 3 (-23.0%, *P*<0.001), 7 (-17.7%, *P*<0.001) and 11 months of age (-14.5%, *P*<0.01) (main effect of genotype: *F*_1,31_ = 57.3, *P*<0.001, Fig 1B). Time spent in REM sleep during the dark phase was also significantly reduced at 3 (-2.4%, *P*<0.001) and 7 months of age (-2.6%, *P*<0.001), whereas REM sleep was not significantly altered at 11 months (*P* = 0.07) (main effect of genotype: *F*_1,31_ = 33.4, *P*<0.001, Fig 1B). Sleep-wake architecture is known to undergo alterations with age in mice, with certain inbred strains showing an initial increase in sleep time followed by a decrease at advanced ages [37]. The increase in wakefulness observed during the dark phase in TgCRND8 in comparison to NTg was more pronounced at 3 months (+25.4%) than at 7 (+20.3%) and 11 months (+15.8%). This may be due to age-dependent changes within TgCRND8 and/or NTg. Potential age-dependent effects were assessed by two-way ANOVA, however, we found no significant differences across age groups within either NTg or TgCRND8 mice for time spent awake (main effect of age, *F*_2,31_ = 0.023, *P* = 0.98), in NREM (main effect of age, *F*_2,31_ = 0.17, *P* = 0.84) or in REM sleep (main effect of age, *F*_2,31_ = 2.77, *P* = 0.08).

During the light phase (i.e, resting phase in mice), TgCRND8 mice spent significantly more time awake at 3 (+5.5%, *P*<0.05), 7 (+5.6%, *P*<0.05) and 11 months of age (+10%, *P*<0.01) (main effect of genotype: *F*_1,31_ = 24.2, *P*<0.001, Fig 1C). Increased wakefulness was accompanied by a significant decrease in NREM sleep at 3 (-6.2%, *P*<0.05), 7 (-5.7%, *P*<0.05) and 11 (-7%, *P*<0.05) months of age (main effect of genotype: *F*_1,31_ = 17.5, *P*<0.001, Fig 1C). When the hourly duration of REM was analyzed, a significant decrease in REM sleep was observed in 11-month-old TgCRND8 mice in comparison to NTg at 9h00 (*P*<0.05), 11h00 (*P*<0.05) and 15h00 (*P*<0.01) (main effect of genotype: *F*_1,23_ = 10.0, *P*<0.05, Fig 1A). However, we found that the total time spent in REM sleep during the light phase did not differ significantly between TgCRND8 and NTg mice at all ages studied (main effect of genotype: *F*_1,31_ = 1.2, *P* = 0.29, genotype x age interaction: *F*_2,31_ = 2.9, *P* = 0.07, Fig 1C). Furthermore, as observed during the dark phase, while a significant effect of genotype on time spent awake and time spent in NREM sleep was apparent, no significant differences across age groups were detected during the light phase within either NTg or TgCRND8 mice for time spent awake (main effect of age, *F*_2,31_ = 1.30, *P* = 0.29), in NREM (main effect of age, *F*_2,31_ = 0.61, *P* = 0.55), or in REM sleep (main effect of age, *F*_2,31_ = 3.19, *P* = 0.05).

In addition to the overall increase in total time spent awake and a decrease in total time spent in NREM sleep across the light-dark cycle (Fig 1), further analysis of sleep-wake cycle parameters supported a phenotype of hyperarousal in TgCRND8 mice. TgCRND8 mice displayed extended bouts of wakefulness as expressed by a significant increase in the average duration of waking episodes at 3 months during the dark (*P*<0.001) and light phase (*P*<0.001) and 11 months during the light phase (*P*<0.01) (DP main effect of genotype: *F*_(1,31)_ = 11.41, *P*<0.01, LP main effect of genotype: *F*_(1,31)_ = 36.4, *P*<0.001, Table 1). In accordance with extended periods of wakefulness, 3-month-old TgCRND8 mice also showed a significant decrease in the number of transitions to NREM sleep during both the dark phase (*P*<0.01) and light phase (*P*<0.01) (DP main effect of genotype: *F*_1,31_ = 15.8, *P*<0.001, LP main effect of genotype: *F*_1,31_ = 12.5, *P*<0.01, Table 1). Furthermore, despite a decrease in the total time spent in NREM sleep (Fig 1), the average duration of NREM sleep episodes was preserved in TgCRND8 mice in comparison to NTg across the light-dark cycle, at all ages studied (DP main effect of genotype: *F*_(1,31)_ = 3.8, *P*>0.05, LP main effect of genotype: *F*_(1,31)_ = 2.8, *P*>0.05, Table 1). Finally, while the average duration of REM sleep episodes was found to be unchanged in TgCRND8 mice during the light and dark phases, a significant decrease in the number of transitions to REM sleep was observed at 3 (*P*<0.01) and 7 (*P*<0.01) months of age during the dark phase (main effect of genotype: *F*_(1,31)_ = 20.6, *P*<0.001, Table 1).

**Table 1:** Average episode duration and number of transitions to wake, NREM sleep and REM sleep during the dark phase (DP) and light phase (LP) in TgCRND8 and NTg mice.

		NTG (3)	Tg (3)	NTG (7)	Tg (7)	NTG (11)	Tg (11)
**Wake duration (s)**	**DP**	162.3 ± 34.2	744.9 ± 187.4***	160.2 ± 47.9	382.7 ± 54.9	182.7 ± 53.1	428.0 ± 98.5
	**LP**	52.6 ± 5.4	91.3 ± 4.0***	48.0 ± 6.2	63.1 ± 5.5	62.8 ± 6.9	95.0 ± 6.6**
**NREM duration (s)**	**DP**	78.3 ± 10.8	60.0 ± 6.0	63.4 ± 8.9	38.0 ± 6.0	75.7 ± 6.7	80.5 ± 8.6
	**LP**	89.1 ± 13.5	114.8 ± 5.8	71.6 ± 8.1	73.3 ± 5.7	101.8 ± 10.0	112.8 ± 5.2
**REM duration (s)**	**DP**	69.9 ± 6.4	65.0 ± 5.3	63.4 ± 3.5	76.0 ± 15.2	57.7 ± 2.8	63.8 ± 6.5
	**LP**	72.6 ± 5.4	68.5 ± 4.0	61.4 ± 6.2	68.0 ± 4.5	68.2 ± 10.3	61.3 ± 10.8
**Transitions to wake**	**DP**	211.3 ± 40.0	75.0 ± 16.1**	232.0 ± 50.2	158.7 ± 25.8	184.3 ± 29.0	81.8 ± 12.6*
	**LP**	310.7 ± 38.1	195.6 ± 6.8**	345.0 ± 33.3	301.6 ± 21.9	246.7 ± 20.1	189.0 ± 6.6
**Transitions to NREM**	**DP**	210.9 ± 39.8	74.5 ± 16.1**	232.4 ± 50.5	158.0 ± 26.0	184.7 ± 28.9	82.0 ± 12.5
	**LP**	310.3 ± 38.0	194.3 ± 7.0**	345.4 ± 33.3	297.9 ± 22.2	246.5 ± 19.7	189.0 ± 6.6
**Transitions to REM**	**DP**	23.7 ± 6.5	8.0 ± 2.6**	25.6 ± 5.1	6.4 ± 1.3**	15.3 ± 2.4	5.3 ± 1.3
	**LP**	42.1 ± 5.0	48.1 ± 5.3	56.8 ± 7.6	50.7 ± 9.0	51.3 ± 9.4	29.3 ± 5.7

Mean values ± SEM. Two-way ANOVAs followed by Tukey’s post-hoc.

* *P*<0.05

** *P*<0.01

***P<0.001.

### TgCRND8 mice show alterations in brain oscillatory activity during wakefulness, NREM and REM sleep

**Fig 2:**
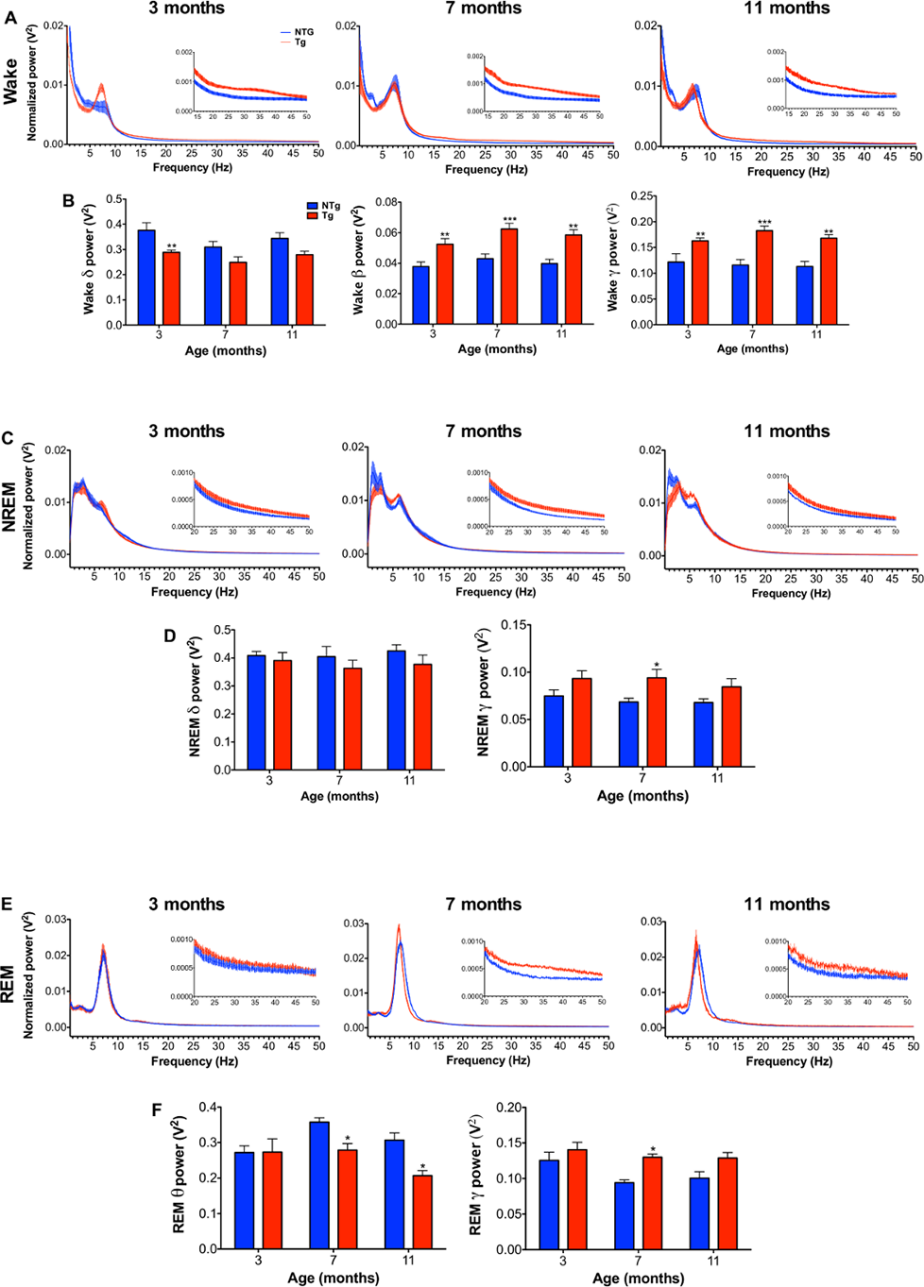
TgCRND8 mice show alterations in brain oscillatory activity during wakefulness, NREM and REM sleep. Normalized power spectrums from 0.125–50 Hz during wakefulness **(A)**, NREM **(C)**, and REM sleep **(E)**, and normalized spectral power quantifications of key frequency bands during wakefulness **(B)**, NREM (**D**) and REM sleep **(F)** at 3 (NTg n = 7, Tg n = 8), 7 (NTg n = 5, Tg n = 7) and 11 months (NTg n = 6, Tg n = 4). (**A** and **B**) During wakefulness, 3-month-old TgCRND8 show a reduction in delta power (0.5–4.5 Hz), and at all ages, higher beta (14–20 Hz) and low gamma power (20–50 Hz) in comparison to NTg. (**C** and **D**) at all ages studied, NREM sleep delta power (0.5–4.5 Hz) was preserved in TgCRND8 in comparison to NTg. Seven-month-old TgCRND8 mice showed higher NREM low gamma power (20–50 Hz) in comparison to NTg. (**E** and **F**) The REM sleep power spectrum was unaltered in 3-month-old TgCRND8 mice, while 7 and 11-month-old TgCRND8 showed reduced REM sleep theta power (7–10 Hz) in comparison to NTg. Seven-month-old TgCRND8 also show higher REM sleep low gamma power (20–50 Hz) in comparison to NTg. The data represented above include vigilance state spectral power analysis of the dark phase (i.e. active phase) for wakefulness and of the light phase (i.e. resting phase) for NREM and REM sleep. Error bars represent SEM. Two-way ANOVA, followed by Tukey’s post-hoc test. * *P*<0.05, ** *P*<0.01, *** *P*<0.001.

Different vigilance states give rise to changes in brain oscillatory activity whereby fast frequency oscillations in the beta and gamma range are indicative of cortical activation and wakefulness, while NREM sleep is characterized by slow frequency delta oscillations, and REM sleep by fast frequency oscillations with predominant theta activity [38]. Brain oscillatory activity during wakefulness, NREM sleep and REM sleep is known to be altered in AD patients [8, 39] and changes have also been reported in AD mouse models [40–43]. Specifically, human studies support an overall slowing of the EEG in AD during wakefulness and REM sleep, comprising an increase in delta and theta power and a decrease in alpha and beta power [39]. The majority of studies in AD mouse models have reported alterations in the EEG power spectrum that do not recapitulate the profile of EEG slowing observed in AD patients and may even suggest an opposite pattern of spectra power alterations involving a shift towards higher frequency oscillation [40, 42, 43]. In order to characterize brain oscillatory activity during wakefulness, NREM sleep and REM sleep in the TgCRND8 model of AD, spectral power analysis of EEG activity during these three vigilance states was conducted at 3, 7 and 11 months of age during the dark phase and light phase (Fig 2).

Consistent with a phenotype of hyperarousal, the most prominent change in cortical activity observed in TgCRND8 mice was a shift in the waking power spectrum towards fast frequency oscillations in the beta and gamma range across the light-dark cycle. Specifically, beta power (14–20 Hz) was significantly higher in TgCRND8 than in NTg at 3 (DP: *P*<0.01, LP: *P*<0.01), 7 (DP: *P*<0.001, LP: *P*<0.01) and 11 months (DP: *P*<0.01, LP: *P*<0.01) (DP main effect of genotype: *F*_1,30_ = 36.40, *P*<0.001, Fig 2B, LP main effect of genotype: *F*_1,31_ = 36.2, *P*<0.001, data not shown). In addition, heightened low gamma power (20–50 Hz) was observed at 3 (DP: *P*<0.01, LP: *P*<0.01), 7 (DP: *P*<0.001, LP: *P*<0.001) and 11 months (DP: *P*<0.01, LP: *P*<0.01) (DP main effect of genotype: *F*_1,31_ = 36.80, *P*<0.001 Fig 2B, LP main effect of genotype: *F*_1,31_ = 39.40, *P*<0.001 data not shown). Concomitant with higher power of fast frequency oscillations during wakefulness, a reduction in the power of slow oscillations in the delta range (0.5–4.5 Hz) was observed in TgCRND8 in comparison to NTg during the dark phase at 3 months (*P*<0.01) and during the light phase at 3 (*P*<0.01), 7 (*P*<0.05) and 11 months of age (*P*<0.01) (DP main effect of genotype: *F*_1,31_ = 14.01, *P*<0.001 Fig 2B, LP main effect of genotype: *F*_1,31_ = 27.33, *P*<0.001 data not shown).

Despite a decrease in the total time spent in NREM sleep (Fig 1), NREM delta power (0.5–4.5 Hz) was preserved across all ages during the light and dark phase in TgCRND8. However, NREM low gamma power (20–50 Hz) was higher in TgCRND8 compared to NTg during the dark phase (*P*<0.01) and light phase (*P*<0.05) at 7 months of age (DP main effect of genotype: *F*_(1,31)_ = 13.6, *P*<0.001, data not shown, LP main effect of genotype: *F*_(1,31)_ = 10.5, *P*<0.01, Fig 2D) Interestingly, the REM sleep power spectrum was preserved at 3 months of age across the light phase and dark phase, with alterations emerging at the more advanced pathological stages studied. At this later time point, we detected a significant reduction in REM sleep theta power (7–10 Hz) during the light phase in 7 (*P*<0.05) and 11-month-old TgCRND8 (*P*<0.05) (main effect of genotype: *F*_1,31_ = 7.6, *P*<0.01, Fig 2F) and during the dark phase in 11-month-old TgCRND8 (*P*<0.05) (main effect of genotype: *F*_(1,30)_ = 6.0, *P*<0.05, data not shown). Furthermore, REM sleep low gamma power (20–50 Hz) was higher in TgCRND8 than in NTg at 7 months during both the light phase (*P*<0.05) and the dark phase (*P*<0.05) (DP main effect of genotype: *F*_(1,30)_ = 7.3, *P*<0.05, data not shown, LP main effect of genotype: *F*_(1,31)_ = 12, *P*<0.01, Fig 2F).

### 3-month-old TgCRND8 mice differ from NTg in their homeostatic response to total sleep deprivation

TgCRND8 mice display sleep-wake cycle dysfunction characterized by increased total time spent awake and decreased total time spent in NREM sleep across the light-dark cycle. Homeostatic sleep drive is a critical factor regulating the sleep-wake cycle. In order to assess whether the homeostatic process of sleep regulation is altered in TgCRND8 mice, 3-month-old NTg and TgCRND8 mice were subjected to 6 hours of TSD, and their sleep recovery response during the 2-hour period immediately following TSD was studied. Given that the profile of sleep-wake cycle disruption did not differ significantly between TgCRND8 age groups under baseline conditions (Fig 1), the homeostatic sleep response was tested in the 3-month-old group only.

**Fig 3:**
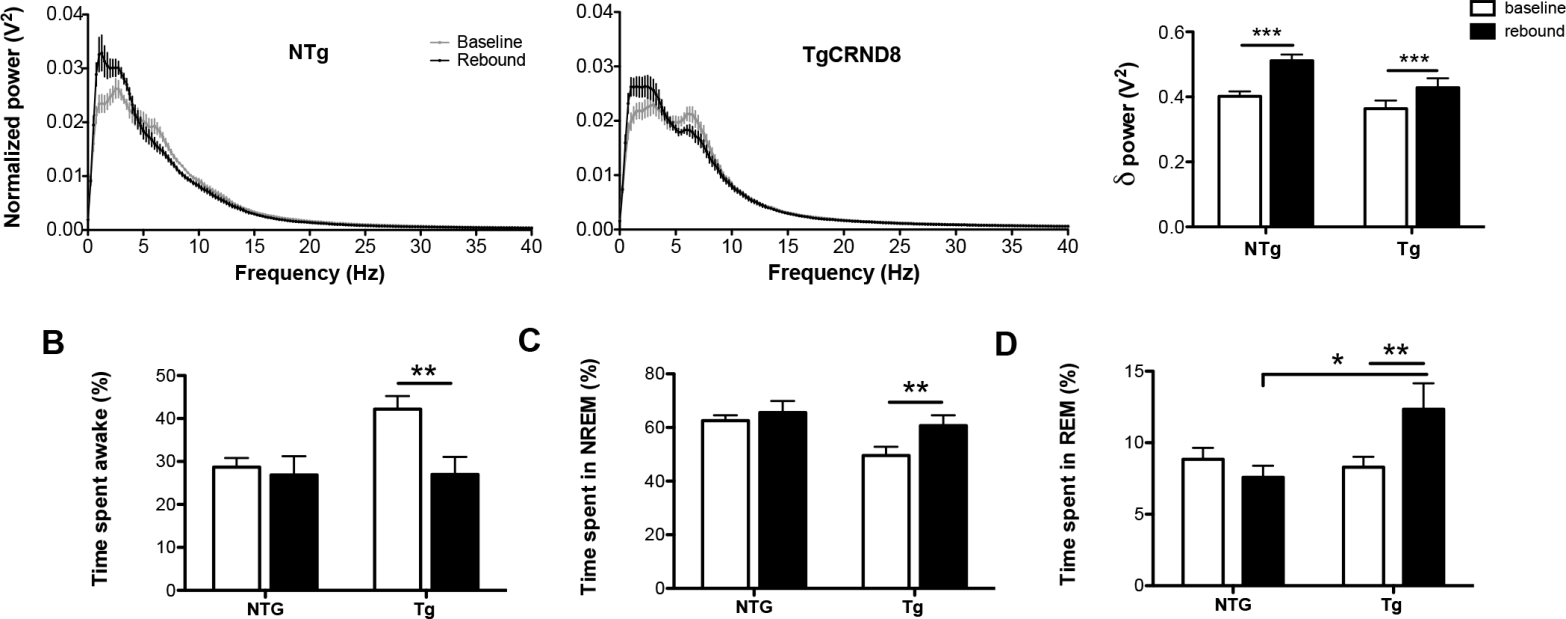
Three-month-old TgCRND8 mice differ from NTg in their homeostatic response to total sleep deprivation. Normalized NREM sleep power spectrums from 0.125–40 Hz and quantification of NREM delta power (0.5–4.5 Hz) **(A)**, time spent awake **(B)**, time spent in NREM sleep **(C),** and time spent in REM sleep **(D)** under baseline and rebound conditions in 3-month-old NTg (n = 7) and TgCRND8 mice (n = 9). Total sleep deprivation (TSD) was performed for 6 hours, beginning at the onset of the light phase. The rebound period corresponds to the 2-hour period immediately following TSD. (**A)** Both TgCRND8 and NTg mice show a significant shift towards higher NREM delta power during the rebound period in comparison to baseline. Interestingly, the NREM delta power rebound response was blunted in TgCRND8 mice, with an 18% and 27% increase between baseline and rebound in TgCRND8 and NTg, respectively. (**B)** TgCRND8 show a significant decrease in time spent awake during the rebound period in comparison to baseline. No significant change in time spent awake during the rebound period in comparison to the baseline period was observed in NTg, and this response did not differ significantly from TgCRND8. (**C)** TgCRND8 show a significant increase in time spent in NREM sleep during the rebound period in comparison to baseline. No significant change in time spent in NREM sleep during the rebound period in comparison to the baseline was observed in NTg, and this response did not differ significantly from that observed in TgCRND8. (**D)** TgCRND8 show a significant increase in time spent in REM sleep during the rebound period in comparison to baseline. No significant change in time spent in REM sleep during the rebound period in comparison to the baseline period in NTg, and this response differed significantly from that observed in TgCRND8. Time spent in REM sleep during the rebound period was significantly greater in TgCRND8 in comparison to NTg. Error bars represent SEM. Mixed design two-way ANOVAs followed by simple effects analysis or Tukey’s post hoc where appropriate, * *P*<0.05, ** *P*<0.01, *** *P*<0.001.

During the 6 hour sleep deprivation period (8:00 AM-2:00 PM), both groups spent >96% of the period awake. Specifically, TgCRND8 spent 96.72% awake, 3.22% in NREM and .06% in REM, while NTg mice spent 97.83% awake, 2.17% in NREM and 0% in REM. Time spent awake, in NREM or in REM during the sleep deprivation period was not significantly different between TgCRND8 and NTg (*P*>0.05 for all comparisons, two-tailed, unpaired student’s t-test, data not shown). Following total sleep deprivation, a homeostatic rebound response was observed in both NTg (*P*<0.001) and TgCRND8 mice (*P*<0.001), as demonstrated by a significant shift towards higher NREM sleep delta power during the rebound period in comparison to baseline (genotype x time period interaction: *F*1,14 = 19.9, *P*<0.001, Fig 3A). Interestingly, the NREM delta power rebound response was blunted in TgCRND8 mice, with NTg and TgCRND8 mice displaying a 27% and 18% change from baseline, respectively (Fig 3A). TgCRND8 mice also showed a significant increase in time spent in NREM (*P*<0.01) (main effect of time period: *F*1,14 = 6.7 *P*<0.05, Fig 3C) alongside a decrease in time spent awake during the rebound period (*P*<0.01) (main effect of time: *F*1,14 = 7.64, *P*<0.05, Fig 3B), responses that did not differ significantly from that observed in NTg. Furthermore, TgCRND8 mice showed a homeostatic increase in time spent in REM sleep during rebound (*P*<0.01), which was significantly greater than that observed in NTg (genotype x time period interaction: F1,14 = 7.5, *P*<0.05, Fig 3D). Notably, despite a homeostatic shift towards higher NREM sleep delta power (Fig 3A), NTg mice did not show a significant increase in NREM (Fig 3C) or REM sleep time (Fig 3D) between baseline and rebound.

### Quantification of total Aβ_42_ within key brain regions of sleep-wake cycle regulation

Amyloidosis progresses differentially across distinct brain regions. Although immunohistochemical studies have shown that the cortex and hippocampus are among the first regions affected by plaque deposition in TgCRND8 mice [34], little is known about the progression of amyloidosis within key regions regulating sleep-wake states in this mouse model. We therefore performed a region-specific quantification of total Aβ_42_ levels from the prefrontal cortex, thalamus, hypothalamus, and brainstem in 3, 7 and 11 month-old TgCRND8 mice.

**Fig 4:**
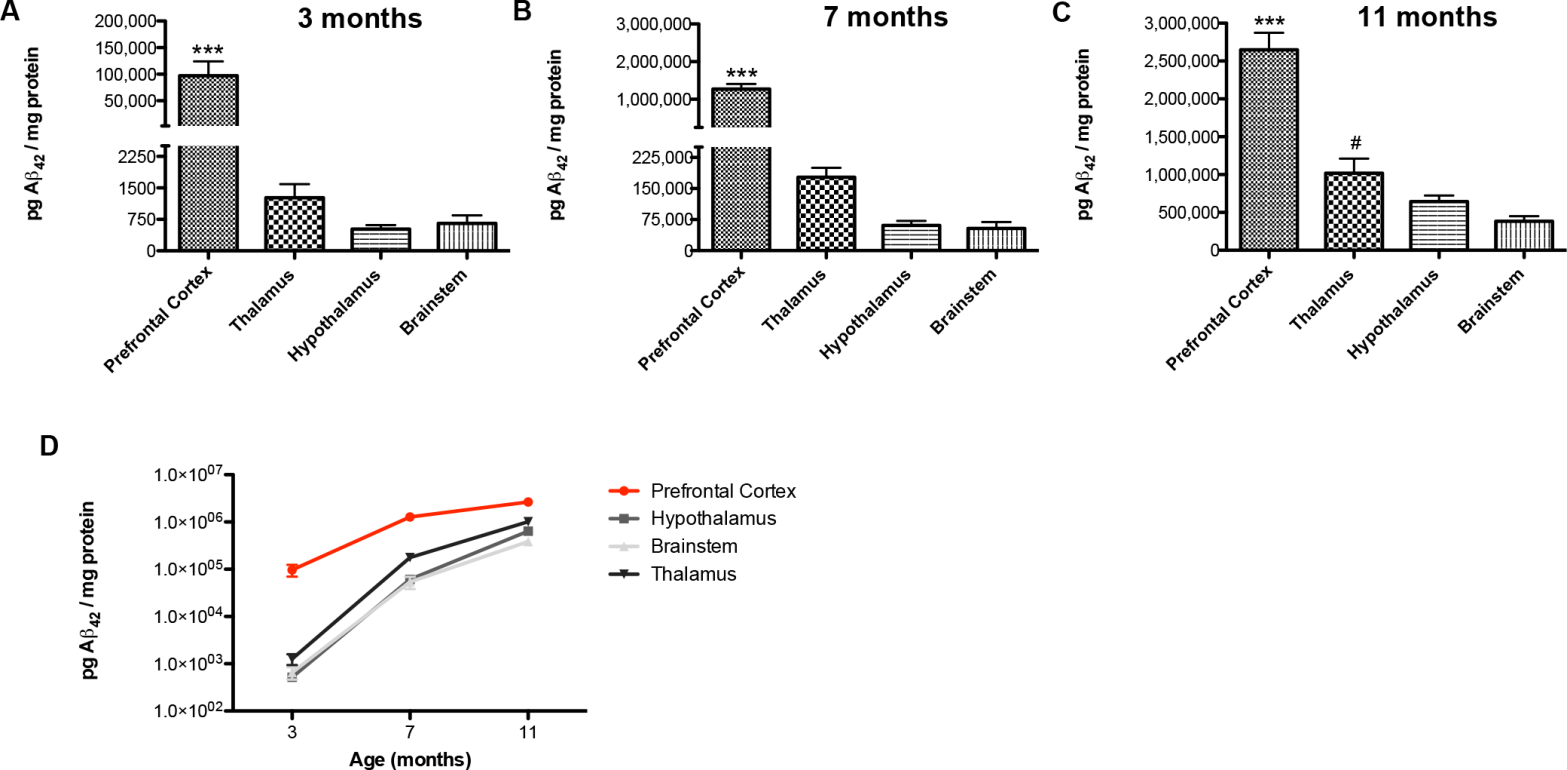
Quantification of Aβ_42_ levels from key regions regulating the sleep-wake cycle. Quantification of total Aβ_**42**_ levels from the prefrontal cortex, hypothalamus, thalamus and brainstem of 3 (n = 7), 7 (n = 9) and 11-month-old (n = 7) TgCRND8 mice. Picograms (pg) of total Aβ_**42**_ are normalized to milligrams (mg) of protein per sample. (**A, B and C)** At 3, 7 and 11 months of age the prefrontal cortex contains the highest level of total Aβ_**42**_, differing significantly from the hypothalamus, thalamus and brainstem. (**C)** At 11 months of age, the thalamus contains significantly higher total Aβ_**42**_ than the brainstem. (**D)** Progression of total Aβ_**42**_ overexpression in the prefrontal cortex, hypothalamus, thalamus and brainstem at 3, 7 and 11 months of age. Error bars represent SEM. Fig **4A**, **4B** and **4C** were analyzed by one-way ANOVA for the effect of brain region at a given age, followed by Tukey’s post-hoc. * Denotes a significant difference between the prefrontal cortex and each other region. # Denotes a significant difference between the thalamus and the brainstem. */^#^ *P*<0.05, ** *P*<0.01, *** *P*<0.001.

At all ages studied the prefrontal cortex contained the highest levels of total Aβ_42_ in comparison to the thalamus, brainstem and hypothalamus (*P*<0.001 for all regions, Fig 4A, 4B and 4C). Already at 3 months of age, total Aβ_42_ levels in the prefrontal cortex were 76-, 148- and 186-fold greater than the thalamus, brainstem and hypothalamus, respectively (Fig 4D). Within the subcortical regions studied, the thalamus contained the highest levels of total Aβ_42,_ and reached statistical significance in comparison to the brainstem at 11 months (*P*<0.05, Fig 4C). By 7 months of age, total Aβ_42_ levels in the thalamus reached the order of magnitude observed in the prefrontal cortex at 3 months, while Aβ_42_ levels in the hypothalamus and brainstem reached such magnitude at 11 months (Fig 4D).

### Prazosin, a selective α_1_-adrenergic receptor antagonist, differentially affects sleep in 3.5-month-old NTg and TgCRND8 mice

**Fig 5:**
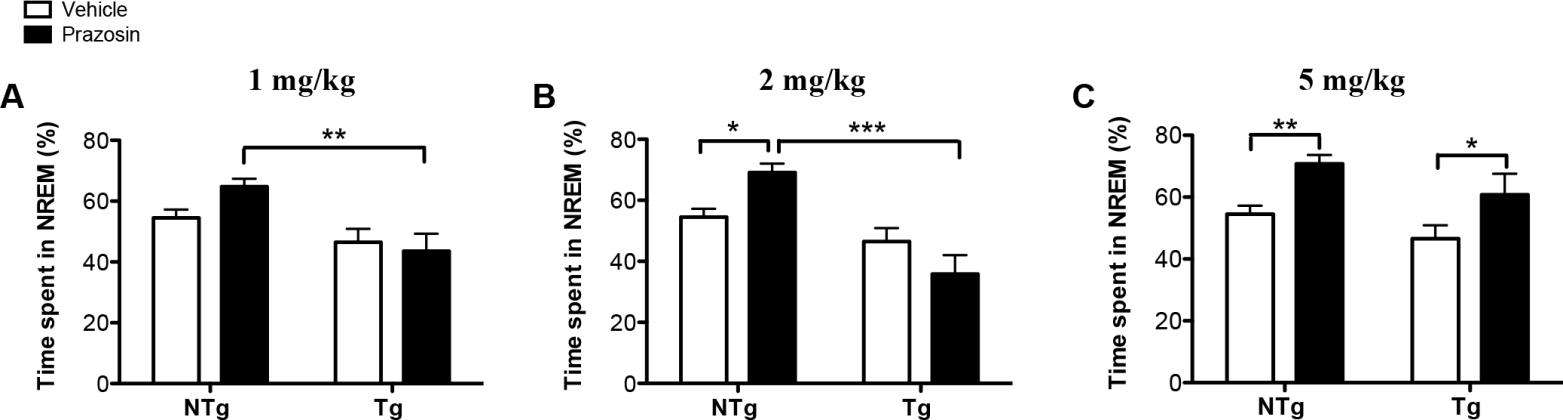
Prazosin differentially affects NREM sleep in 3.5-month-old TgCRND8 and NTg mice. Time spent in NREM sleep during the 2-hour period following administration of the α_**1**_-adrenergic antagonist, prazosin, at 1 (**A**), 2 (**B**) and 5 (**C**) mg/kg versus vehicle in 3.5-month-old NTg and TgCRND8 mice. Prazosin or vehicle was administered at 10:00 AM via an intraperitoneal injection. (**A)** Treatment with Prazosin at 1 mg/kg does not significantly affect the percent time spent in NREM sleep when compared to vehicle in both NTg and TgCRND8. (**B)** At 2 mg/kg, treatment with Prazosin significantly increases the time spent in NREM sleep in comparison to vehicle in NTg mice only. The observed increase in NREM sleep in NTg following treatment with 2 mg/kg prazosin differed significantly from time spent in NREM sleep following treatment with 2 mg/kg in TgCRND8. (**C)** At 5 mg/kg, prazosin significantly increases time spent in NREM sleep in both NTg and TgCRND8 mice. Error bars represent SEM. NTg vehicle n = 11, NTg 1, 2 and 5 mg/kg n = 8, Tg vehicle n = 11, Tg 1 and 2 mg/kg n = 9, Tg 5 mg/kg n = 6. Fig **5A**, **5B** and **5C** were analyzed by individual two-way ANOVAs comparing each prazosin dose to vehicle, followed by Tukey’s post-hoc or simple effects analysis where appropriate, * *P*<0.05, ** *P*<0.01, *** *P*<0.001.

Potential mechanisms underlying the phenotype of hyperarousal in TgCRND8 mice include increased activity within one or more of the wake-promoting neurotransmitter systems. One such candidate is the noradrenergic system, which exerts potent wake-promoting effects through post-synaptic α_1_- and β-adrenergic receptors [44]. Interestingly, in AD patients, evidence supports a compensatory increase in noradrenergic activity in response to noradrenergic cell loss [30–32]. Given the arousal promoting properties of noradrenaline, such compensatory alterations in noradrenergic activity could disrupt the sleep-wake cycle towards a hyperaroused state. We therefore tested whether noradrenergic blockade with the selective α_1_-noradrenergic receptor antagonist, prazosin, could normalize sleep-wake patterns in TgCRND8 mice. Prazosin readily penetrates the blood brain barrier and is centrally active [24]. 3.5-month-old NTg and TgCRND8 mice were treated with I.P injections of 1, 2 and 5 mg/kg of prazosin at the beginning of the light phase and vigilance state durations for the two-hour period following treatment were quantified (Fig 5).

Analysis of the first 2 hours following prazosin administration at 1, 2 and 5 mg/kg in TgCRND8 and NTg revealed a dose-dependent effect on NREM sleep in both genotypes. Interestingly, we found that an increase in NREM sleep was observed in TgCRND8 mice only at the highest dose tested, 5 mg/kg (Fig 5C), whereas NTg mice responded with an increase in NREM sleep at both 2 mg/kg (Fig 5B) and 5 mg/kg (Fig 5C). Specifically, at 1 mg/kg, treatment with prazosin did not significantly affect the time spent in NREM sleep in either genotypes (main effect of dose: *F*_1,35_ = 0.80, *P*>0.05, Fig 5A). Whereas at 2 mg/kg, effects on the time spent in NREM sleep emerged in NTg (*P*<0.05) but not in TgCRND8 (*P*>0.05) (genotype x dose interaction: *F*_1,35_ = 8.20, *P*<0.01, Fig 5B). Following treatment with 5 mg/kg of prazosin, time spent in NREM sleep was significantly increased in both NTg (*P*<0.01) and TgCRND8 (*P*<0.05) (main effect of dose: *F*_1,32_ = 12.96, *P*<0.01, Fig 5C). Prazosin was also shown to decrease time spent in REM sleep following treatment at 2 mg/kg in NTg (*P*<0.01) and TgCRND8 (*P*<0.05) (main effect of dose: *F*_1,35_ = 13.14, *P*<0.001, Table 2) as well as following treatment with 5 mg/kg in NTg (*P*<0.001) but not in TgCRND8 (*P*>0.05) (main effect of dose: *F*_1,31_ = 21.29, *P*<0.001, Table 2). Finally, time spent awake was not significantly affected by prazosin in either TgCRND8 or NTg mice at 1 mg/kg (main effect of dose: *F*_1,35_ = 0.19, *P*>0.05, Table 2), 2 mg/kg (dose x genotype interaction: *F*_1,35_ = 4.60, *P*<0.05, simple effects for the effect of dose on genotype: *P*>0.05 for NTg and TgCRND8, Table 2) or 5 mg/kg (main effect of dose: *F*_1,32_ = 6.69, *P*<0.05, Tukey’s test for the effect of dose on genotype: *P*>0.05 for NTg and TgCRND8, Table 2).

**Table 2:** Duration of wake, NREM sleep and REM sleep during the 2 hours following prazosin administration in 3.5-month-old TgCRND8 and NTg mice.

		Wake (%)	NREM (%)	REM (%)
**Vehicle**	**NTg**	40.2	54.5	5.3
	**Tg**	48.1	46.5	5.4
**1 mg/kg**	**NTg**	32.5	64.8	2.7
	**Tg**	51.8^**##**^	43.6^**##**^	4.3
**2 mg/kg**	**NTg**	32.9	69.1*	2.2*
	**Tg**	61.1^**###**^	35.8^**###**^	3.0*
**5 mg/kg**	**NTg**	28.6	70.7**	0.6***
	**Tg**	35.5	60.7*	3.7^**#**^

Mean values ± SEM. Individual two-way ANOVAs, comparing each dose to vehicle, followed by Tukey’s post-hoc.

# and * signify between and within genotype effects, respectively.

*^/#^ *P*<0.05

**^/##^ *P*<0.01

***^/###^ *P*<0.001.

## Discussion

### Sleep-wake cycle architecture

The TgCRND8 mouse model of AD exhibits pronounced sleep-wake cycle dysfunction, characterized by hyperarousal across the light phase (resting phase) and dark phase (active phase). Disrupted sleep-wake patterns were already apparent at the early stages of plaque deposition, and included increased total time spent awake and decreased total time spent in NREM sleep during the resting and active phase. Interestingly, TgCRND8 mice did not show any alterations in the average duration of NREM sleep episodes or in baseline NREM sleep delta power, a marker of NREM sleep intensity. These findings suggest efficient sleep maintenance, despite an overall decrease in total time spent in NREM sleep. Furthermore, a shift in the waking power spectrum towards fast frequency beta (14–20 Hz) and low gamma (20–50 Hz) oscillations was apparent across the light-dark cycle at all ages studied, and a significant increase in the average duration of waking episodes alongside a decrease in the number of transitions to NREM sleep was present at 3 months. Taken together, these findings suggest a state of hyperarousal, which may in turn contribute to dysfunctional sleep initiation in TgCRND8 mice giving rise to the overall decrease in total time spent in NREM.

In humans, aging is associated with the emergence of sleep disturbances that include a decrease in total sleep time, reduced slow-wave sleep, reduced REM sleep, increased sleep fragmentation, and increased daytime sleepiness [45]. The same sleep disturbances manifest in AD patients but are more pronounced than in aged controls. Furthermore, the magnitude of sleep disturbances in AD patients increases as the disease progresses [46]. In order to assess the progression of sleep disturbances with advancing pathology and age in TgCRND8, the sleep-wake cycle was studied at three time-points in NTg and TgCRND8 mice. During the active phase, the increase in wakefulness observed in TgCRND8 was more pronounced at 3 months of age (+25%), than at 7 (+20.3%) and 11 months (+15.8%). These findings may relate to changes in sleep-wake patterns across age groups in NTg and/or TgCRND8 mice. However, analysis of age-dependent effects on time spent in wake, NREM or REM sleep revealed no significant differences across age in either NTg or TgCRND8 mice during the active phase. During the resting phase, the increase in wakefulness between NTg and TgCRND8 was more pronounced at the more advanced age studied (+10%) than at 7 (+5.6%) or 3 months (5.5%). This pattern is the reverse of that observed during the active phase and may indicate a worsening of the resting phase sleep deficit at advanced stages in TgCRND8. Furthermore, a trend towards a decrease in the total time spent in REM sleep between 11 month-old NTg and TgCRND8 was apparent during the light phase and appeared to be due to an age-dependent decrease in REM in TgCRND8. However, as observed during the active phase, no significant differences in time spent awake, in NREM or in REM sleep were observed across age in the NTg or TgCRND8 group. It is possible that significant age-dependent changes in sleep/wake duration may emerge at more advanced ages than those assessed in the current study. To this effect, when age-related changes in sleep were studied at 3, 6, 12, and 22 months in C57BL/6 mice, one of the strains that forms the hybrid background used in the current study, a significant effect of age on sleep/wake duration was only observed in the 22 month-old group [37].

The homeostatic sleep process is a driving force regulating the sleep-wake cycle, whereby sleep loss generates a compensatory increase in sleep duration and intensity, the latter indexed by NREM sleep delta power [21, 22]. A deficit in homeostatic sleep drive in TgCRND8 mice could potentially contribute to the phenotype of hyperarousal observed in TgCRND8, whereby a lack of homeostatic pressure would result in frequent wakefulness and poor sleep quality. In order to investigate the homeostatic process of sleep regulation in TgCRND8 mice, we subjected 3-month-old NTg and TgCRND8 mice to total sleep deprivation and studied their rebound response. We found that TgCRND8 mice showed a significant homeostatic increase in sleep time (NREM and REM) and decrease in wakefulness during the rebound period, while NTg mice did not show a significant change in wake, NREM or REM time during rebound. This difference in homeostatic response between TgCRND8 and NTg was statistically significant for time spent in REM sleep during rebound. These findings may arise due to the fact that TgCRND8 mice spend significantly less time asleep during the light phase than NTg and are therefore already sleep deprived, per se, under baseline conditions. The amount of experimental sleep deprivation required to induce a significant increase in sleep time and decrease in wakefulness may therefore be lower in TgCRND8 than in NTg mice, as TgCRND8 may be closer to the threshold level that stimulates a homeostatic response. In addition, it is important to note that although NTg mice did not show a rebound increase in sleep time, NTg mice did display the characteristic increase in NREM sleep delta power that occurs following sleep deprivation. This finding, along with the fact that both NTg and TgCRND8 mice spent >96% of the 6-hour sleep deprivation period awake, results that did not differ between genotypes, supports effective sleep deprivation in both groups. Furthermore, while TgCRND8 mice display a homeostatic increase in sleep time during rebound, the “quality” or intensity of rebound sleep in TgCRND8, assessed by NREM delta power, is altered compared to NTg. This is supported by our findings that although both NTg and TgCRND8 show a significant homeostatic shift towards higher NREM delta power during the rebound period, this effect was blunted in TgCRND8. Therefore, although a significant rebound effect was observed in TgCRND8 mice for the duration of sleep, the homeostatic process may not be fully functional in these mice, possibly due to increased noradrenergic drive.

The observed phenotype of reduced NREM sleep and increased wakefulness during both the resting and active phase in TgCRND8 mice partially replicates alterations observed in AD patients. While AD patients experience increased wakefulness and a decrease in slow-wave NREM sleep during the night [8, 47], a finding replicated during the respective resting phase of TgCRND8 mice, excessive sleepiness often occurs during the day in AD [48–50]. In contrast, TgCRND8 mice show increased wakefulness during their active phase, rather than increased sleepiness. Furthermore, the observed increase in wakefulness and reduction in NREM sleep in TgCRND8 mice is more pronounced during the active phase than during the resting phase. It may be expected that dysfunctional sleep regulation would preferentially affect sleep during the resting phase, where time spent asleep predominates. However, if the mechanisms leading to sleep-wake cycle disruption involve hyperactivity within wake-promoting circuits, rather than a deficit in sleep promotion, it is logical that the phenotype may be more pronounced during the phase where wake-promoting circuits are predominantly active (i.e. the active phase). Furthermore, although AD patients can experience excessive daytime sleepiness, such differences have been shown to occur in the first half of the day [48], whereas sundowning (i.e., anxiety, agitation, pacing, wandering) can occur in the late afternoon or evening [51, 52]. Increased wakefulness during the active phase in TgCRND8 mice, may therefore relate to activity patterns observed during the late afternoon or evening in AD patients. Finally, it is important to consider the polyphasic nature of sleep consolidation in mice, whereby, sleep occurs in short bouts (< 5 minutes) across the resting and active phase [53]. It is therefore likely that although they may share underlying mechanisms, AD-associated sleep disturbances may manifest themselves differently over 24 hours in AD mouse models and AD patients.

At mild stages of the disease, AD patients do not experience a reduction in REM sleep whereas at moderate to severe stages, a reduction in REM sleep has been shown to emerge [47, 54]. Interestingly, our study showed that although total time spent in REM in TgCRND8 mice was significantly reduced at 3 and 7 months during the active phase, total time spent in REM sleep was preserved during the resting phase at all ages studied. The REM sleep deficit during the active phase may therefore be secondary to the pronounced decrease in NREM rather than an intrinsic dysfunction of REM sleep regulation. Furthermore, the REM sleep power spectrum did not differ from NTg mice at 3 months of age during both the resting and active phases, while a decrease in REM sleep theta power was present at 7 and 11 months of age during the resting phase and at 11 months during the active phase, suggesting a gradual disruption of REM sleep at more advanced pathological stages. REM sleep patterns in TgCRND8 mice may therefore reflect the mild stages of AD when REM sleep duration is still intact. This may be explained by the progression of amyloidosis across subcortical regions in TgCRND8 mice, whereby the brainstem, containing key REM sleep controlling nuclei [55–58], only develops plaques at approximately 8 months of age [34]. Furthermore, quantification of total Aβ_42_ from key regions of sleep-wake cycle regulation in the current study revealed lower levels of Aβ_42_ within the brainstem at 3 months of age followed by a pronounced rise at 7 and 11 months.

An important factor to consider when relating the sleep-wake cycle alterations observed in TgCRND8 mice to those present in AD patients is that, as with most mouse models of AD, TgCRND8 mice do not express the complete profile of AD-associated neuropathological features, which in addition to amyloidosis, include neurofibrillary tangle formation and neurodegeneration. There is a great deal of variation in the observed phenotype of sleep-wake cycle alterations across different mouse models of AD, which is likely related to the different transgenes expressed and subsequent neuropathology in these mice. For instance, as observed in TgCRND8 mice, an increase in wakefulness and decrease in NREM sleep has been reported in AD mouse models expressing more than one AD-associated transgene. These include transgenic mice co-expressing mutations in the amyloid precursor protein (APP) and presenilin 1 genes (PSEN1) [43, 59, 60], as well as mice expressing mutant tau in addition to mutant APP and PSEN1 [41]. However, in two studies investigating mouse models that only express one mutant form of the APP transgene, REM sleep time appeared to be preferentially affected over NREM sleep time [42, 61]. Both the PDAPP and Tg2576 APP transgenic models showed preserved NREM sleep time alongside a decrease in REM sleep time at pre-plaque stages. At post-plaque stages, NREM sleep time remained intact in Tg2576 mice [42], while PDAPP mice showed a decrease in active phase NREM sleep [61]. TgCRND8 mice co-express the Swedish and Indiana APP mutations, which are the transgenes individually expressed in the Tg2576 and PDAPP mice, respectively. Considering that the phenotype of sleep disturbances observed in TgCRND8, comprising a NREM sleep deficit, resembles more closely that observed in other multi-transgenic AD mouse models, rather than the early REM sleep deficit observed in the APP transgenic Tg2576 and PDAPP mice, it may be that the severity of neuropathology incurred by the expression of multiple transgenes, rather than the specific transgenes expressed is more closely related to sleep-wake disruption. Furthermore, within the models presenting increased wakefulness and decreased NREM sleep such as TgCRND8, APPswe/PSEN1dE9 [60], APPswe/PSEN1A246E [43] and PLB1-triple [41], there is considerable variability in the patterns of these alterations across the light-dark cycle. As previously discussed, the NREM sleep deficit observed in TgCRND8 mice is more prominent during the active phase than during the resting phase, an effect that is also observed in the APPswe/PSEN1A246E mouse model [43]. Conversely, the APPswe/PSEN1dE9 mouse model, expressing a different mutant form of PSEN1, displays an increase in wakefulness and decreased NREM and REM sleep that is more prominent during the resting phase than the active phase [60]. Furthermore, as seen in TgCRND8 mice, these mouse models do not show the characteristic daytime sleepiness reported in AD patients. Thus far, only the 3xTg mouse model of AD, which expresses APP, PSEN1 and tau mutations, has shown increased resting phase activity alongside decreased active phase activity, reminiscent of the nocturnal awakenings and daytime sleepiness observed in AD patients [62]. However, only circadian activity and not sleep-wake patterns were assessed in the 3xTg mice. Nonetheless, AD mice expressing both Aβ plaques and neurofibrillary tangles, such as 3xTg mice may provide a more comprehensive model of sleep disturbances in AD.

In AD, the hallmark EEG feature during wakefulness and REM sleep is an overall slowing of the EEG, including an increase in delta and theta power along with a decrease in alpha and beta power [39]. In contrast, we observed, a shift in the power spectrum towards faster frequencies in the beta (14–20 Hz) and low gamma range (20–50 Hz). EEG slowing in AD has been hypothesized to be due to functional disconnections between cortical regions as a result of neurodegeneration [39]. The profile of oscillatory activity observed during wakefulness in TgCRND8 may be due to the fact that this AD model represents an earlier stage of amyloid pathology, preceding severe neurodegeneration, since it is well-known that APP mice do not replicate the extent of neuronal loss observed in the brains of AD patients [63]. Interestingly, spectral power changes in both the Tg2576 [40, 42], and the APPswe/PSEN1A246E [43] are not in-line with the characteristic EEG slowing reported in AD patients, but rather involve a shift in power towards fast-frequency oscillations, similar to our observations in TgCRND8. Conversely, the PLB1-triple mouse model of AD, expressing, APP, Tau and PSEN1 transgenes, shows a profile of oscillatory changes that includes an increase in delta power, and resembles EEG slowing present in AD patients [41].

### Quantification of Aβ_42_ within key regions of sleep-wake cycle regulation

Quantification of total Aβ_42_ from the prefrontal cortex, thalamus, hypothalamus and brainstem in TgCRND8 revealed a differential progression of amyloidosis, whereby subcortical, sleep-regulatory regions are less affected by Aβ_42_ accumulation than the prefrontal cortex. At 3 months, total Aβ_42_ levels in the prefrontal cortex were, 76-, 186- and 148-fold higher than those measured from the thalamus, hypothalamus and brainstem, respectively. However, by 7 months, the thalamus contained Aβ_42_ levels on the same order of magnitude observed in the prefrontal cortex at 3 months, while the hypothalamus and brainstem reached such levels at 11 months. Our results are in line with previous immunohistochemical studies that have reported the appearance of plaques as early as 2 months of age in the cortex, while the thalamus and brainstem develop plaques later on, at ~4 and ~8 months respectively [34].

### Effects of α_1_-adrenoreceptor blockade on NREM sleep in 3.5-month-old TgCRND8 and NTg mice

It is known that there is an association between activation of the ascending arousal system of the brainstem and gamma activity [64, 65] and synchrony [64]. It may therefore be plausible that increased activity within one or more of the neurotransmitter systems of the ascending arousal system in TgCRND8 may underlie both the increase in wakefulness and heightened low gamma power (20–50 Hz) observed in these mice. One candidate is the noradrenergic projections of the locus coeruleus (LC). Interestingly, there is evidence for a compensatory increase in noradrenergic tone in AD patients, comprising increased activity at noradrenergic terminals, alterations in adrenoreceptor expression, and sprouting of LC axonal projections to the hippocampus and prefrontal cortex in response to degeneration of the LC [30–33, 66, 67]. In agreement with this, alterations of the noradrenergic system in TgCRND8 include a decrease in the tissue levels of noradrenaline in the hippocampus, concomitant with an increase in tissue normetanephrine, a metabolite of noradrenaline, suggesting a compensatory increase in neurotransmitter release from noradrenergic terminals [68]. Furthermore, our results suggest reduced sensitivity to noradrenergic blockade in 3-month-old TgCRND8 mice. We found that NTg mice showed a 14.6% increase in time spent in NREM sleep following treatment with 2 mg/kg while TgCRND8 mice were not significantly affected by this dose. However, at a higher dose of 5 mg/kg, NREM sleep was increased by 14.2% in TgCRND8 and by 16.2% in NTg mice. The finding that a higher dose of prazosin (5 mg/kg) was required to achieve the same percent increase in NREM sleep as that observed in NTg mice at a lower dose (2mg/kg) may support the notion that increased noradrenergic tone contributes to sleep deficits in 3.5-month-old TgCRND8 mice. This could potentially be mediated by an increase in α_1_-adrenoreceptor expression and/or neurotransmitter release at noradrenergic terminal sites within the ascending arousal system such as the lateral hypothalamus, basal forebrain, laterodorsal /pedunculopontine tegmental nuclei, and/or dorsal raphe nucleus. In turn, a higher concentration of prazosin would be required to promote NREM sleep, through the blockade of α_1_-adrenoreceptor, within these regions. It would therefore be of interest to investigate α_1_-adrenoreceptor expression within noradrenergic terminal fields of the ascending arousal system and to measure functional levels of noradrenaline via microdialysis to assess the state of noradrenergic transmission in TgCRND8.

The current study provides evidence that Aβ overexpression and deposition in the absence of neurofibrillary tangle formation and substantial neurodegeneration is associated with the appearance of sleep-wake cycle alterations in the TgCRND8 mouse model of AD. These findings highlight the role of Aβ in the generation of AD-associated sleep-wake cycle disturbances. A potential role for altered noradrenergic transmission in the generation of these symptoms was suggested here, but will require further studies into the state of the noradrenergic system in TgCRND8 mice.

## Supporting Information

S1 Dataset Episode Duration and Number of Transitions for Wake, NREM and REM Dataset S1. W: wake, NR: non-rapid eye movement sleep, R: rapid eye movement sleep, NTg: non-transgenic, Tg: Transgenic.
(XLSX)

S2 Dataset Total Duration of Wake, NREM and REM Dataset S2. NTg: non-transgenic, Tg: transgenic.
(XLSX)

S3 Dataset Power Spectrums for Wake, NREM and REM Dataset S3. NTg: non-transgenic, Tg: transgenic.
(XLSX)

S4 Dataset Sleep Deprivation Dataset S4. NTg: non-transgenic, Tg: transgenic.
(XLSX)

S5 Dataset Quantification of Aβ_42_ Dataset S5. P: prefrontal cortex, H: hypothalamus, T: thalamus, B: brainstem, C/Cb: cerebellum, mo: months.
(XLSX)

S6 Dataset Prazosin Treatment Dataset S6. NTg: non-transgenic, Tg: transgenic.
(XLSX)

## Abbreviations

AD,: Alzheimer’s disease
APP,: Amyloid precursor protein
Aβ,: Beta-Amyloid
DP,: Dark phase
EEG,: Electroencephalogram
EMG,: Electromyogram
LP,: Light phase
LC,: Locus Coeruleus
NREM,: Non-rapid eye movement
NTg,: Non-transgenic
PSEN-1,: Presenilin-1
REM,: Rapid eye movement
TSD,: Total sleep deprivation
